# Enhancing the magnetic response on polycrystalline nanoframes through mechanical deformation

**DOI:** 10.1038/s41598-022-09647-2

**Published:** 2022-04-08

**Authors:** Mario Castro, Samuel E. Baltazar, Javier Rojas-Nunez, Eduardo Bringa, Felipe J. Valencia, Sebastian Allende

**Affiliations:** 1 Universidad de Santiago de Chile (USACH), Facultad de Ciencia, Departamento de Física, Santiago, Chile; 2 Departamento de Computación e Industrias, Facultad de Ciencias de la Ingeniería, Universidad Católica del Maule, Talca 3480112, Chile, Universidad Católica del Maule, Talca, Chile; 3 Universidad de Santiago de Chile (USACH), Centro para el Desarrollo de la Nanociencia y la Nanotecnología (CEDENNA), Santiago, Chile; 4 CONICET and Facultad de Ingeniería, Universidad de Mendoza, 5500 Mendoza, Argentina; 5 Centro de Nanotecnología Aplicada, Facultad de Ciencias, Universidad Mayor, Santiago, Chile

**Keywords:** Materials science, Nanoscience and technology, Physics

## Abstract

The mechanical and magnetic properties of polycrystalline nanoframes were investigated using atomistic molecular dynamics and micromagnetic simulations. The magneto-mechanical response of Fe hollow-like nanocubes was addressed by uniaxial compression carried out by nanoindentation. Our results show that the deformation of a nanoframe is dominated at lower strains by the compression of the nanostructure due to filament bending. This leads to the nanoframe twisting perpendicular to the indentation direction for larger indentation depths. Bending and twisting reduce stress concentration and, at the same time, increase coercivity. This unexpected increase of the coercivity occurs because the mechanical deformation changes the cubic shape of the nanoframe, which in turn drives the system to more stable magnetic states. A coercivity increase of almost 100 mT is found for strains close to 0.03, which are within the elastic regime of the Fe nanoframe. Coercivity then decreases at larger strains. However, in all cases, the coercivity is higher than for the undeformed nanoframe. These results can help in the design of new magnetic devices where mechanical deformation can be used as a primary tool to tailor the magnetic response on nanoscale solids.

## Introduction

Mechanical loads affecting magnetic properties have been a very promising technique to tailor the magnetic response of macro to nanoscale solids. For instance, plastically deformed materials under torsional stress have shown a significant increase in coercivity compared with the pristine sample. The increase in coercivity is justified with decrease in the exchange energy in the neighborhood of the defects, by the production of grain boundary structure, and/or by an increase in dislocation density in the grain boundary structure^1–4^. Other approximations, as the case of flexible material have shown promising results to control magnetic behavior by means of purely elastic deformation^5,6^. In this aspect, hard magnetic soft materials, typically hard-magnetic nanoparticles embedded in flexible polymeric materials, are very convenient structures to induce fast shape change, external induced actuation, and huge elastic deformations^7,8^. Of course, the magnetic contribution is limited to the fraction of magnetic particles embedded in the soft matrix, where a large concentration could limit the flexible behavior of this kind of devices.

The search for new nanoscale structures with porous structures has attracted attention due to technological applications such as magnetic storage, biomedical treatments, smart switches, and so on^9–12^. These structures consider several morphologies or architectures, with a remarkable surface to volume ratio, which can introduce unexpected behavior in nanoscale solids. These particles can be synthesized in several shapes such as squares, cubes, and hollow systems^13,14^, among others. One of these novel structures is a nanoframe, a hollow nanocube, mainly synthesized with a template approach from inorganic materials like metals and magnetic systems^15^. Regarding to their magnetic properties, nanoframes have attracted increasing interest since they display size dependent magnetic response^16^ .

To the date metallic nanoframes have shown outstanding optical^17^, magnetic^18^, and electrochemical^16^ properties. For instance, the magnetization of 2D square nanoframes was studied by micromagnetic simulations^19^. Different magnetization reversal processes were obtained when the system size conditions were varied. Hysteresis loops of Co square nanoframes were modeled and experimentally measured^18^, identifying the effects of the defects in the magnetization steps. In particular, Fe nanoframes have been synthesized by thermolysis with sodium oleate^15^, with an approximate length size of 20 nm. The particular geometry of nanoframes has motivated theoretical studies in the field. Micromagnetic simulations were performed on these systems. If we consider the shape and size scale of nano-objects of Fe^20^, these parameters affect the magnetization reversal and the magnetic anisotropy. 2D squared-like systems were considered, finding different magnetic anisotropies from micromagnetic simulations. For instance, magnetostatic properties were studied in a Fe Kagome symmetry lattice^21^, where the reversal process depends on the shape and geometric parameters of the magnetic arrays.

Metallic nanoframes, due to their novel design can show promissory mechanical behavior, which coupled with their magnetic response can give room to unexpected magnetic behavior. The study of nanoframes as a strain modulated magnetic device is precisely the focus of this contribution. Recently, several studies have investigated the failure resistance of nanolattices and unveiled how to control the surprisingly high elastic regimes^22–24^. Besides these studies, there are still missing atomistic information to unveil the mechanism behind elasto-plastic transition and failure. All in all, molecular dynamics techniques combined with magnetic simulations can offer valuable information on magneto-mechanical response in nano-designed materials. In our case, nanoframes can show unexpected mechanical properties due to the cooperative response of their filament structure. This response can turn on the nanostructure bending, twisting or densify in presence of mechanical strain, leading to shape or topological transformations.

In this work, we report how mechanical strain modifies the magnetic behavior of Fe nanoframes by means of micromagnetic and molecular dynamics simulations (MD). Since magnetic simulations are typically studied in non-strained solids, we assess how the magnetization response of a single 3D Fe nanoframe is modified under mechanical compression, going from elastic to inelastic regimes. This study addresses the stability of magnetic states due to mechanical compression. This stability was observed in an increment of the coercivity at several compressive strains before fracture. These results can help in the design of magnetic devices under mechanical compression for technological applications.

## Results

**Figure 1:**
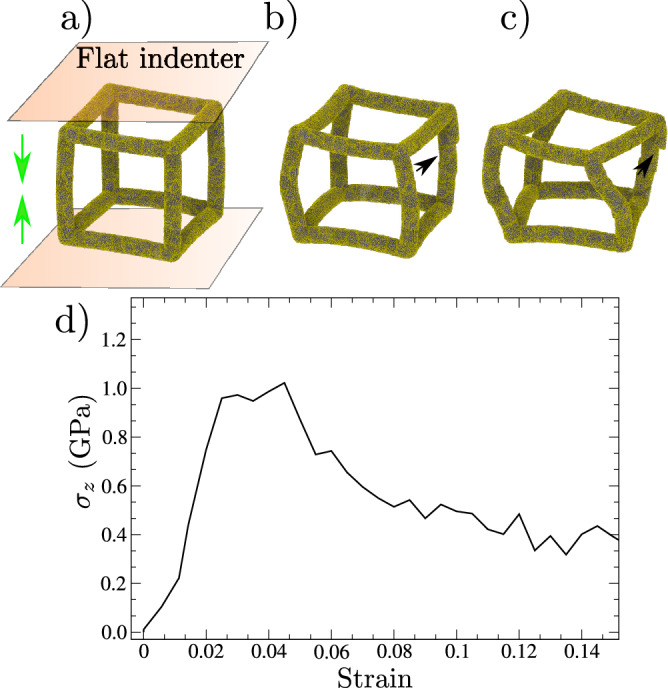
(**a**–**c**) Show snapshots of the deformed nanoframe at strains of 0.0, 0.05, and 0.15, respectively. Transparent planes represent the flat indenter, while the green arrows depict the displacement direction (z-axis) of both planes. Arrows in (**b**) and (**c**) point to grain boundary displacement events. (**d**) Stress–strain curve obtained from the nanoindentation test.

To study the mechanical properties of nanoframes we carried out molecular dynamics simulations. Here, the mechanical strain is introduced by means of a flat indenter pushing upper and lower faces (z-axis) of the nanoframe. To characterize the mechanical behavior, the Fig. 1a shows a typical strain-stress curve obtained from a nanoindentation simulation for different strains. For $\varepsilon =0.0$, the nanoframe shows just a minor curvature which is a consequence of the relaxation process. The radius of curvature is many times larger that the initial frame side. This minor bending can be expected due to the small filament radius, which allows curvature driven by the stresses in the nanoframes corners. At 0.05 strain filament bending increases roughly until the point when relative grain motion is significant, leading to significant grain slip, as pointed by an arrow in Fig. 1b.

Larger stresses not only compress the nanostructure but also twist the frame perpendicular to the indentation direction (Fig. 1c). In Fig. 1d, the stress observed during nanoindentation grows rapidly from 0 to 1.0 GPa, from strains of 0 to 0.05, in line with several MD results using the same interatomic potential^25–27^. It is worth mentioning that after maximum compressive stress, the stress decreases until it reaches roughly constant flow stress beyond $\varepsilon =0.12$. Some simulations of nanoparticles or nanocrystalline surfaces under nanoindentation observe continuous softening at large penetrations depths due to dislocation nucleation and motion^28–30^. Recent simulations and experiments for nanoboxes observe hardening due to dislocation interactions^31^, as in Taylor hardening. Here, the constant flow stress can be attributed to the particular geometry of the nanoframes, where at high bending and twisting reduces the stress concentration and at the same time reduces dislocation nucleation, as will be shown later on.

**Figure 2:**
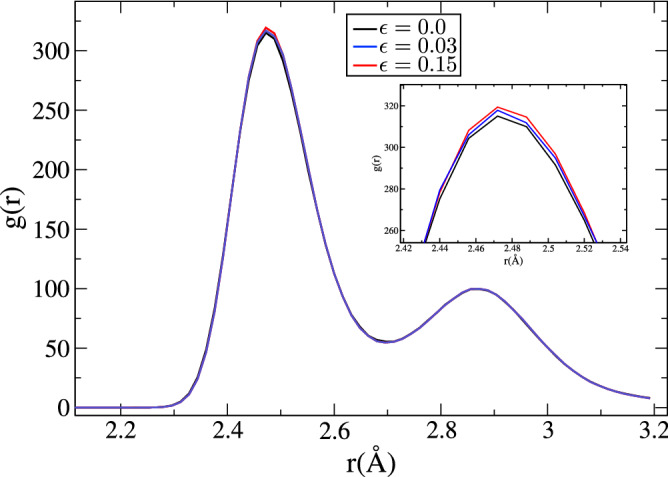
Pair correlation function for strains of 0, 0.03 and 0.15. Inset show a zoom of the g(r) peak.

Since our MD results will be applied to micromagnetic simulations, we inspect several sources which can modify or introduce changes in the magnetic response. The Fig. 2 shows the pair correlation function, *g*(*r*), under strain. The shape and magnitude of *g*(*r*) are practically unaffected during compression, suggesting that the relative positions between the Fe atoms remain constant during the nanoindentation. This result supports the assumption that the magnetic parameters remain constants when strain is $\le 0.15$.

**Figure 3:**
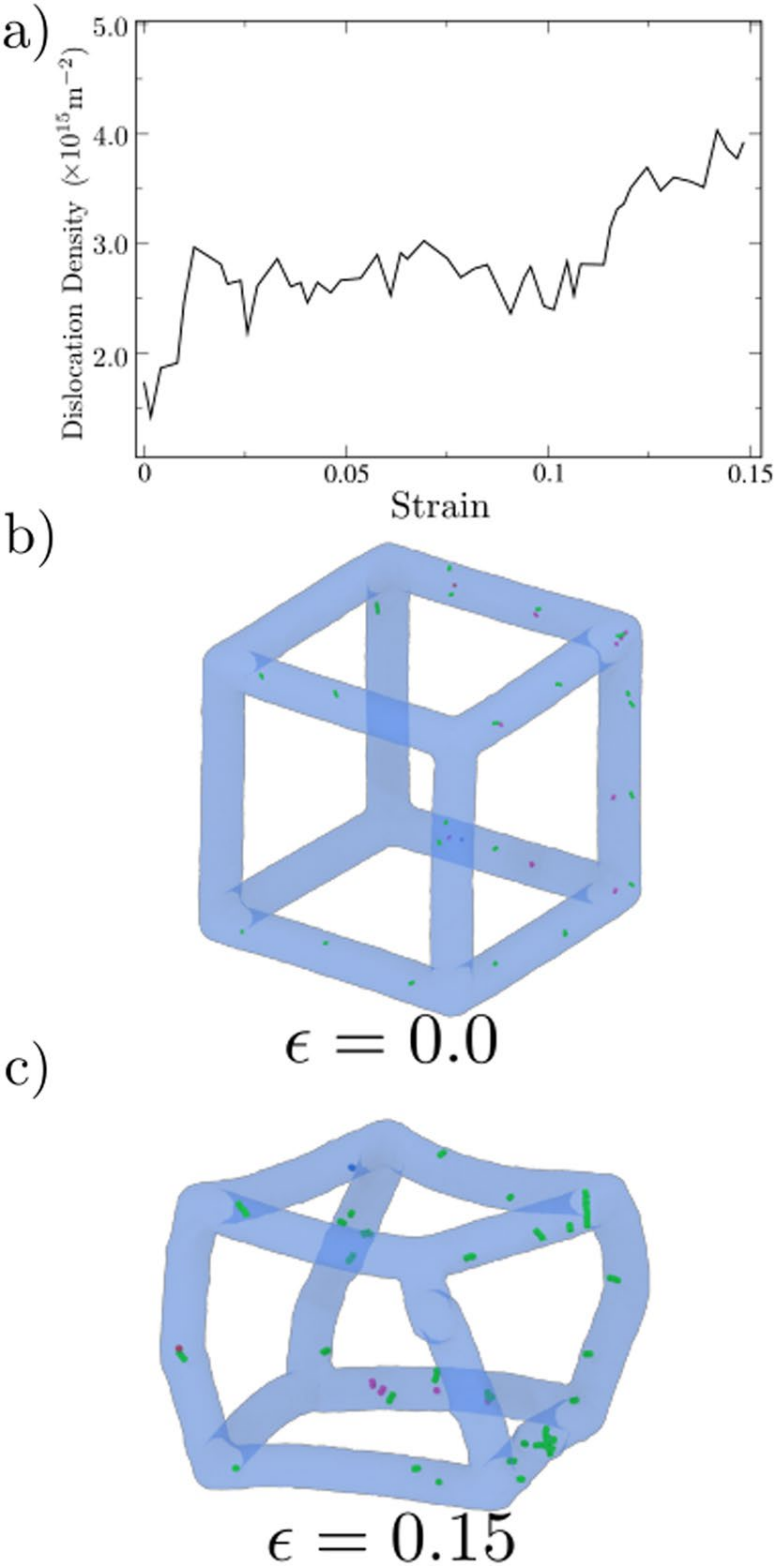
(**a**) Total dislocation density vs. strain. (**b**,**c**) Snapshots showing dislocations. The blue semitransparent region corresponds to the nanoframe surface, while green and pink lines correspond to $1/2\langle 111\rangle$, and $\langle 100\rangle$ dislocations, respectively. (**b**) Zero strain and (**c**) 0.15 strain.

Dislocations will generate a large stress-field, which can change local density and, therefore, local magnetic moments^32^. Figure 3a shows the presence of dislocations even at $\varepsilon =0.0$, but they are detected by the dislocation extraction algorithm (DXA)^33^. These results show dislocation in grain boundaries with no presence of them inside the grains. After an initial rise in dislocation activity, there is almost no change in dislocation density since deformation is accommodated by grain boundary (GB) activity and nanoframe bending and rotation. As the strain increases beyond 0.1, the total dislocation density increases to $\sim$ 4.0$\times 10^{15}$ m$^{-2}$. This increase is attributed to the growth of the small dislocation embryos (Fig. 3b) and also to the nucleation of a few dislocations along the whole nanoframe as shown in Fig. 3c. However, the dislocation found inside the grains does not surpass 2% of the total dislocations density. Besides, dislocations inside the grains are mostly partial dislocations, which nucleate at a GB, travel across the grain, and are absorbed by the opposite grain boundary. Usually, stacking-faults (SF) do not contribute to hardening, but a large density of criss-crossing SF might lead to sessile dislocations, which might display hardening, as it was qualitatively discussed by^31^. Here, dislocation density is very small, nearly a factor of 100 smaller than typical atomistic simulations of deformation of bulk nanocrystaline Fe^25,34^, and there are no dislocation junctions, which would aid hardening. This helps to explain the constant flow stress previously discussed. We also note that for a single nanowire without buckling nor barreling, larger volumetric strains might lead to phase transitions^35^, but in our simulations, volumetric strain is low, thanks to bending of the nanostructure.

Inspecting Fig. 2, we can consider, as a first approximation, that the magnetic parameters would not be affected during the deformation. Similarly, we can consider that the magneto-elastic anisotropy is approximately zero when applying the system strain from 0 to 0.15. This approximation is used because the pair correlation function between these deformations stages do not significantly change, see Fig. 2. The self-magnetostatic interaction of the system is responsible of the magneto-elastic effect in our results.

**Figure 4:**
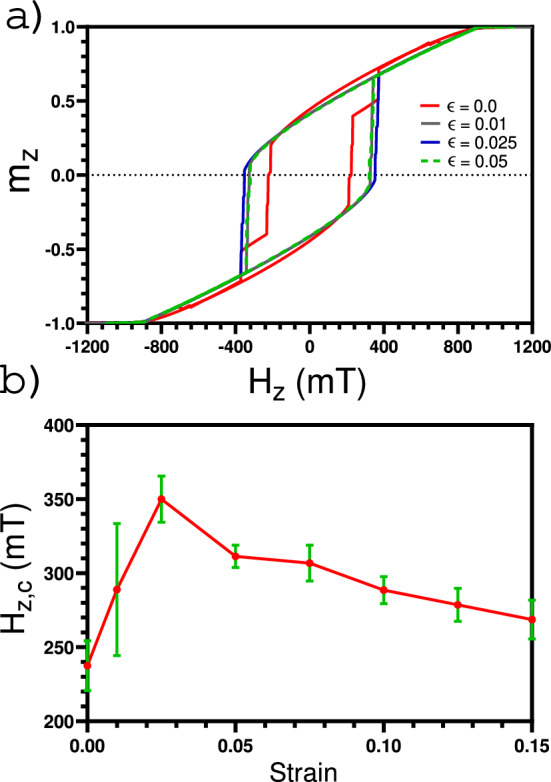
(**a**) Histeresis curve at different strain. (**b**) Coercivity as a function of a strain.

After observing that the magnetic parameters would not be affected, as a first approximation, for strains less than 0.15, we start to study how the magnetic properties change when a nanoframe is subjected to strain. Specifically, we study the coercivity of a nanoframe subjected to a given strain when a magnetic field is applied along the z-axis (i.e., the nanoindentation axis). Figure 4a shows how the hysteresis curve changes when stress is applied to our system. It can be observed that there is an abrupt change of the coercivity when applying any strain to the system. The latter can be better appreciated in Fig. 4b. The error bars show the variation of the coercivity when considering four different MD samples generated with different random seeds. In this figure, one can observe a non-monotonic behavior of the coercivity when strain is increased. It can be noticed that, for small strains, the coercivity increases until it reaches a maximum of about 350 mT for a strain of 0.025, and then the coercivity decrease.

**Figure 5:**
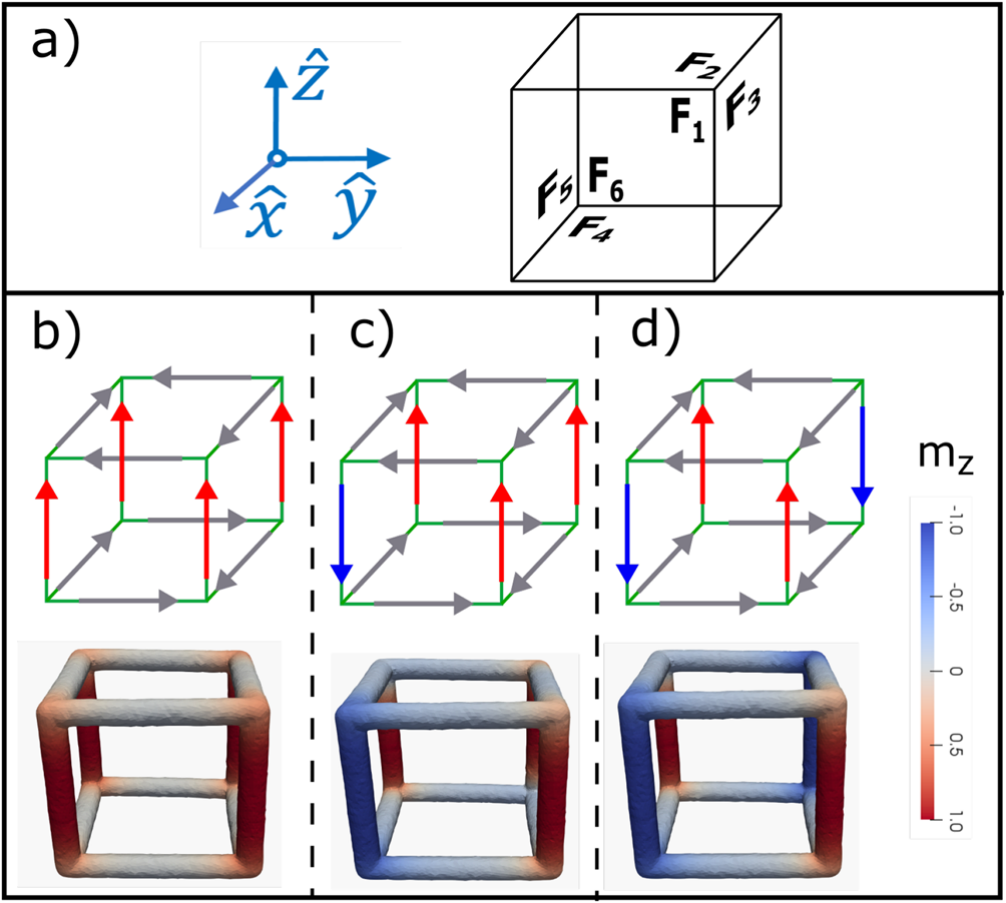
(**a**) Show a reference system for the nanoframe, where F_1_, F_2_, F_3_, F_4_, F_5_ and F_6_ correspond to the cube faces. (**b**) Magnetic reversion of a nanoframe without strain. (**b**–**d**) Show the magnetic profile observed during the magnetic reversion considering magnetic field values of − 80 mT, − 222 mT, and − 226 mT, respectively.

**Figure 6:**
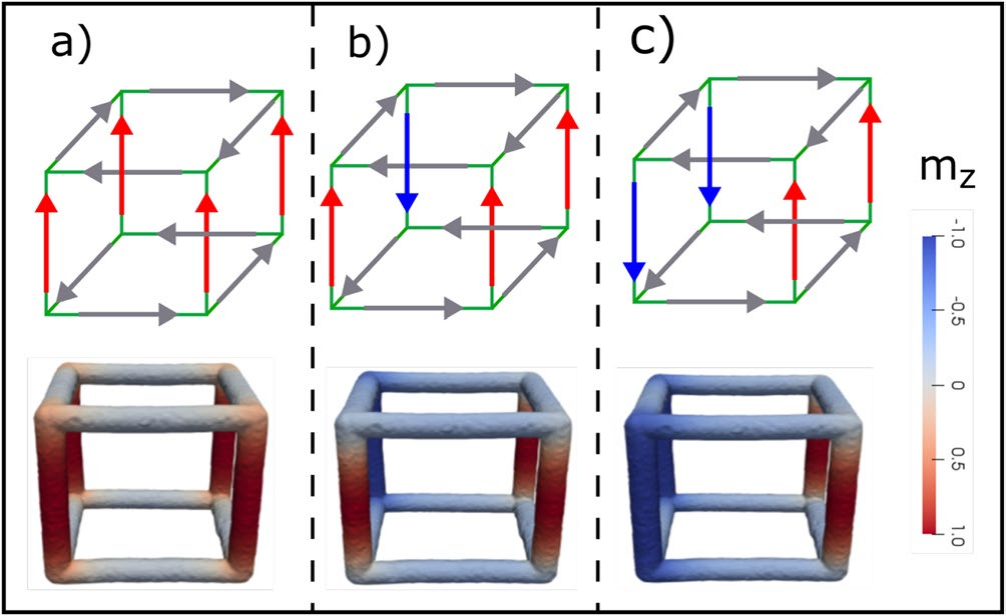
(**a**–**c**) Show the magnetic profile observed during the magnetic reversion considering magnetic field values of − 220 mT, − 358 mT, and − 366 mT, respectively.

The coercivity variation when we applied strain to the system can be understood by analyzing the magnetic reversion of the system. Figures 5 and 6 show the reversion of the magnetization of the nanoframe for a strain of 0 and 0.025, respectively. It is observed that the magnetic reversion in both cases is different. For a visual guide of the magnetic reversion, each face of the nanoframe has been labeled using the nomenclature shown in Fig. 5a, then, if we refer to a specific face for a magnetic vortex, that means that the magnetic moments of the four edges of this face form a magnetic vortex. For the case where there is no strain, it can be observed that the first magnetic moments going into the plane are those at the edges of the base and top of a cube (faces $F_2$ and $F_4$ of Fig. 5b). There are no magnetic vortices formed. As the magnetic field is further reduced, the magnetization reversion starts at the vertical edges, where only one vortex is formed on face $F_1$ (see Fig. 5c,d). It should be noted that the vortex formation reduces the magnetostatic interaction, making the system more stable. On the other hand, in Fig. 6 ($\varepsilon = 0.025$), we can observe that due to the deformation, it is easier for the magnetic moments to form vortices. Initially, the magnetic moments that are at the base and the top of the nanoframe revert, forming vortices, see faces $F_2$ and $F_4$ on Fig. 6a, where the edges of these faces are perpendicular to the magnetic field. As the magnetic field is further reduced, the magnetic moments that form the vertical edges also form magnetic vortices, see faces $F_5$ in Fig. 6b and $F_1$ in Fig. 6c. These magnetic vortices reduce the dipolar interaction of the system, making the system much more stable in comparison with the case of no strain, i.e., the coercivity increases since it is necessary to reduce the magnetic field considerably to remove it from that configuration.

## Conclusions

Together with micromagnetic simulations, atomistic simulations have been used to study the magneto-mechanical response of deformed Fe nanoframes. Molecular Dynamics (MD) simulations of deformation are carried out by nanoindentation using a flat indenter. Magnetic response is studied as a function of the applied strains, using the nanostructures from MD as input. Our main results can be listed as follows:

- Deformation modes of polycrystalline nanoframes respond to the cooperative deformation of their constituents, leading to filament bending.
- Plastic deformation is dominated by grain boundary sliding instead of dislocation activity.
- Filament bending is accompanied by nanoframe twisting perpendicular to the deformation path. The synergy between grain boundary sliding and bending/twisting results into nearly constant flow stress for strains larger than 0.1.
- An increase in coercivity of 100 mT was found for strains smaller than 0.025. The enhanced magnetic activity is attributed to filament bending, which facilitates magnetic vortex formation in the nanoframe.
- Coercivity decreases as the strain increases for values larger than 0.025. This result is because the deformation of the nano-frame modifies the shape anisotropy making the vortices more unstable when an external magnetic field is applied.

We note that our while results and analysis are focused on a single cubic nanoframe; to date there is a broad family of nanoframes such as octahedron, cuboctahedron, trigonal bipyramid, decahedron^36–39^, among others, which might show unexpected magnetic responses under compressive strain. Besides, we hope that future studies might shed light on the strain-modulated response of other nanostructures, including nanolattices^40^, nanofoams^41^ or auxetic materials^42^, where a deformation assisted by densification or filament bending and twisting could enhance or modify the magnetic response on highly porous materials. We believe that the results presented here not only reveals how strain enhance the magnetic properties of a particular nanoporous materials, but also could help in the design of new nanomaterials for applications in new magnetic devices, flexible electronics, or smart switches whose response is mainly modulated by mechanical deformation.

## Methods

**Figure 7:**
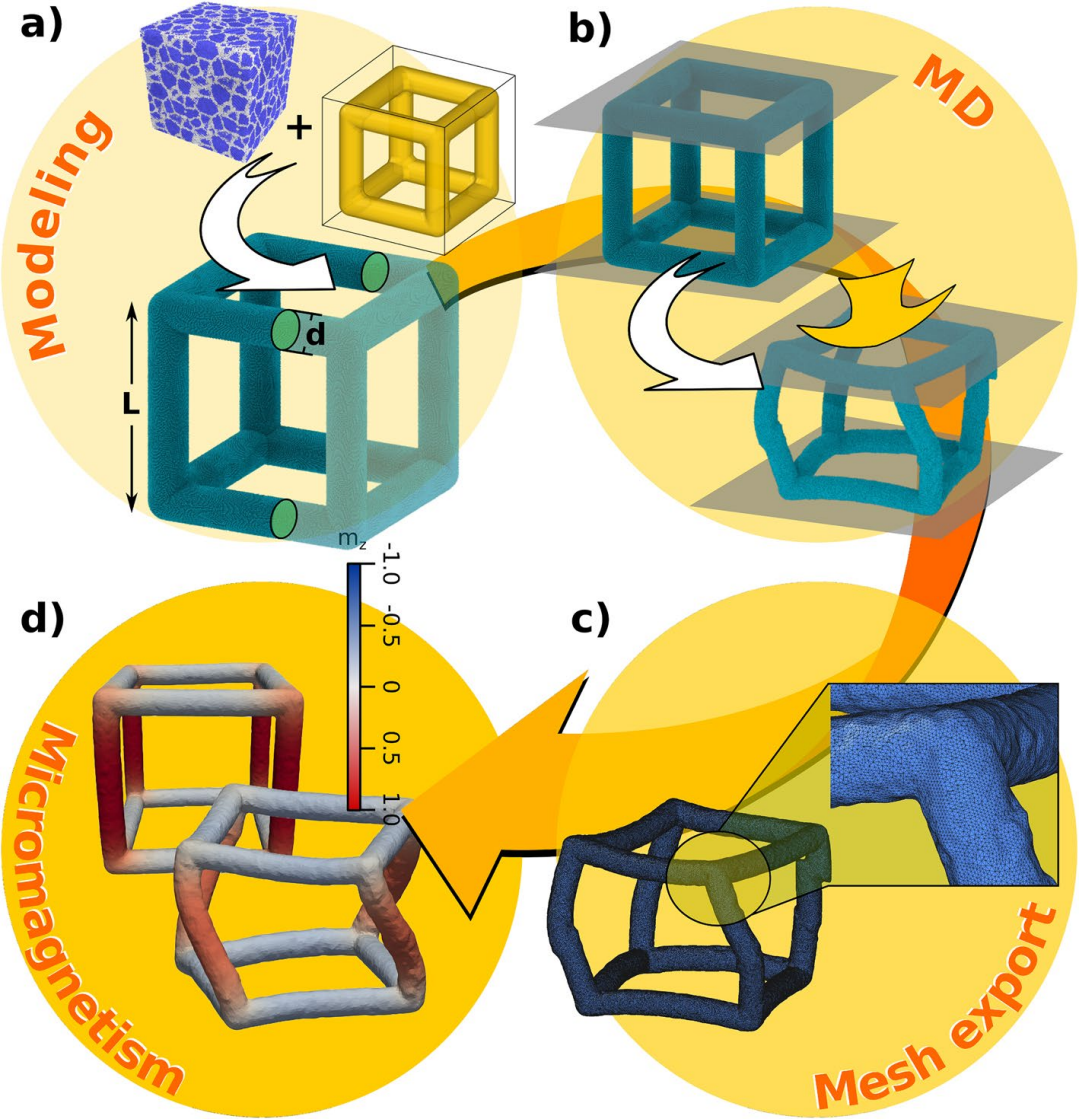
Schematic of methodology to combine (**a**) atomistic designed structures that were (**b**) compressed using molecular dynamics. Then result surface meshes are (**c**) exported to compute (**d**) micromagnetic simulations.

The polycrystalline nanoframes considered in this article are Fe hollow nanocubes schematically represented in Fig. 7. These cubic nanoframes have an edge size (L) of 40 nm and 8 nm thickness (d). The study of the magnetic polycrystalline nanoframes under mechanical compression was done by combining two methods: classical Molecular Dynamics (MD) simulation to study the mechanical compression of the nanoframes, and micromagnetic simulation for the study of the magnetic hysteresis loops for nanoframes at a given compression strain.

### Molecular Dynamics simulation

The nanoframe was modeled using a previously relaxed nanocrystalline Fe supercell with an average grain size of 5 nm. The supercell construction and relaxation procedure are the same used in Ref.^43^, where random crystal orientations are merged using a Voronoi tessellated space. The construction of the nanoframe is performed within a distance of 4 nm from the edges and vertices of a 40 nm edged cube, resulting in a frame with filaments of 8 nm of thickness. To obtain reliable results and statistics, we perform five independent simulations. Each simulation considers a nanoframe of the same size but with a different grain boundary structure. The stochastic nature of the nanoframe is controlled by the Voronoi, which allows to modify the orientation and centroids of the grains, keeping constant the average grain size of each sample.

Atomistic simulations of Fe nanoframes were conducted using the LAMMPS code^44^. The interaction between Fe atoms was simulated using the Ackland potential^26^, which has been successfully used to obtain mechanical properties and phase transformation under high pressure^26,45^. The sample was relaxed at 300 K utilizing a velocity rescale algorithm during 200 fs, using a 1.0 fs timestep. The mechanical behavior of nanoframes was studied through nanoindentantion. The Fe nanoframe is compressed by a flat indenter from both lower and upper regions, using an indenter velocity of 1.0 m/s, as is shown in Fig. 7b . The flat indenter is modeled through an anharmonic potential

$$\begin{aligned} U_i=k(z_i-z_0)^3, \end{aligned}$$

where $z_i$ is the z-coordinate of the atom *i*, $z_0$ the indenter position, $k=20$eV/Å$^3$ a constant which represent the indenter stiffness. Rendering and some analysis were performed using the OVITO code^46^. By strain, we refer to the displacement of the nanoindenter normalized to the nanoframe cube side, which gives some measure of uniaxial strain along the indentation direction for the whole nanostructure. The stress in the indentation direction was obtained through the virial stress tensor:

$$\begin{aligned} \sigma _z=\sum _{i\ne j}\frac{1}{\Omega _i}z_{ij} F_{ij}^z, \end{aligned}$$

where $\Omega _i$ is the atomic volume of the *i*-atom. The terms $F_{ij}^z$ and $z_{ij}$ correspond to the z-component of both, force, and relative position of a pair *i*, *j* interacting particles, respectively. Finally, the search of complex defects as dislocations is carried out with the Dislocation Extraction Algorithm (DXA)^33^ which performs an automated recognition and classification of dislocations in crystalline materials with defects.

### Micromagnetic simulation

Different stages in the molecular dynamics simulations are extracted to simulate their magnetic response. To generate the mesh file that will be used in the micromagnetic simulation, we compute the surface mesh of the atomic configuration using OVITO (for each stage)^46^. Then, the surface mesh is imported into GMSH^47^ to generate the volume mesh with an average distance between two nodes near to 0.8 nm (Fig. 7c). For smaller distances, i.e., if the distance between two nodes is 0.5 nm, we do not observe a variation in the shape of the final magnetic configuration and the value of the coercivity field. The micromagnetic simulations (Fig. 7d) were done using Nmag micromagnetic modeling package^48^. The magnetic material used to simulate the magnetic frame correspond to Fe. In this case, the magnetic parameters are the magnetization saturation $M_s=1.7 \times 10^6$ and the exchange stiffness $A = 2.0 \times 10^{-11}$^49^. Also, a Gilbert damping factor of 0.5 was considered. The exchange length of this material is defined as $\sqrt{2 A/\mu _0 M_s^2}=3.3$ nm. The visualization of the nanoframes was done using ParaView^50^.
